# Supplementary material for: The Many Roads from Alternative Splicing to Cancer: Molecular Mechanisms Involving Driver Genes
Source: Cancers (Basel). 2024 Jun 1;16(11):2123. doi: 10.3390/cancers16112123 (PMC11171328; doi:10.3390/cancers16112123)
Supplement: Supplementary file 1 [file cancers-16-02123-s001.zip › cancers-3020969-supplementary.pdf]

The Many Roads from Alternative Splicing to Cancer. Molecular Mechanisms Involving Driver Genes

Francisco Gimeno-Valiente, Gerardo López-Rodas, Josefa Castillo and Luis Franco

SUPPLEMENTARY MATERIAL

Supplementary Table S1. Cancer driver genes regulated by alternative splicing[1]

Gene	Splicing events[1]	Mechanism[2]	References
TP53	esk, IR, at, ai, ce, me	mcis	[96,191,192]
KRAS	esk, at, a5', ce, me	mcis, ep	[181,182,193–195]
BRAF	esk, a5'	N/R	[196,197]
ANAPC1	esk	mcis	[198]
ARID1A	a3'	ocis	[109,110]
KMT2C	esk, a3'	SF	[120]
PTEN	esk, IR, at	mcis	[97,99,101,192]
NRAS	esk, at, o	N/R	[199,200]
CTNNB1	esk	SF	[121]
CDKN2A	esk, at, ai, a5'	mcis	[104,201–204]
IDH1	IR, at	mcis	[10,205]
RB1	esk	mcis	[206,207]
ATM	esk	mcis	[208]
NF1	esk, ce	mcis	[209]
SMAD4	esk, ai	mcis	[210]
FAT1	IR, ai	N/R	[211]
FBXW7	ai	N/R	[212]
NOTCH1	at	mcis	[213]
EGFR	esk, at	mcis, SF, ep	[122,214–218]
KDM6A	a3'	esk	[219]
ATRX	esk	mcis, o	[220–222]
SETD2	o	mcis	[223]
ERBB2	esk	mcis	[224]
NFE2L2	esk, ai	mcis, o	[34]
CDH1	a3'	mcis, SF	[123]
VHL	esk, ai	SF	[225–227]
KEAP1	esk, ai	N/R	[228,229]
EP300	esk	SF	[124]
PIK3R1	esk	mcis	[201,219]
GATA3	a3'	mcis	[201,221]
AR	at, a3', ce	SF, ep	[179,230–232]
FGFR3	esk, me	N/R	[233–235]
HRAS	esk, a3', a5'	mcis	[181,200,236–238]

BRCA2	esk	mcis	[239–241]
BAP1	a3'	mcis	[221,223]
AKT1	ce	N/R	[242,243]
STK11	esk, at, a3'	mcis	[244,245]
ESR1	esk, at, ai, a3', ce	mcis, ocis	[108,246–249]
PTCH1	esk, ai, ce	mcis, SF	[114,250,251]
NOTCH2	esk, at	mcis	[252,253]
CTCF	esk, ai	N/R	[254]
CASP8	a3'	N/R	[255]
CDK12	a5'	mcis	[256]
KIT	ai, o	mcis	[257–259]
HLA-A	IR	N/R	[260,261]
MYD88	esk, IR, ai, a5'	SF	[125–127]
TCF7L2	esk, a5'	SF	[128,262,263]
AXIN1	esk	N/R	[264]
PPP2R1A	esk, a3', a5'	N/R	[265]
FGFR2	me	SF, ep	[70,79,180,266–268]
ELF3	esk, a5'	mcis	[269]
DNMT3A	esk, at, ai, ce	ep	[270–275]
BCL2	at	N/R	[276]
EZH2	esk, ai, a5', ce	mcis, SF	[79,104,111–113,140,277–280]
ALK	esk, ai	ep	[176,281,282]
IDH2	IR	mcis	[10,283]
RHOA	esk	SF	[284]
F3B1	esk	SF	[285]
MED12	esk	mcis	[286]
TGFBR2	esk	mcis	[287,288]
RUNX1	ai	N/R	[289–291]
MAX	me	SF	[131]
IRF4	esk, at, ai, a3', a5'	SF	[292]
KDR	at	N/R	[293]
SGK1	IR, a3'	mcis	[294]
PRDM1	ai	ep	[167]
CCND1	at	mcis	[295]
NF2	esk	N/R	[296]
MYCN	esk	o	[297,298]
FLT3	esk, a3', a5'	N/R	[252]
RAC1	ce	SF	[133,299]
BRCA1	esk	mcis	[300–302]
RET	at	N/R	[303,304]
MEN1	IR	mcis	[305]
CACNA1D	esk	N/R	[306]
SMAD3	a5'	o	[307]
BCLAF1	ce	SF	[134]
STAT3	a3', o	N/R	[308–310]
ERBB4	esk	N/R	[311,312]
MET	esk	N/R	[313–315]
FN1	esk	SF, o	[316–319]

TET1	at	ep	[175]
IKZF1	esk	N/R	[320,321]
FANCD2	at	SF	[135]
EIF1AX	IR, ce	mcis	[322]
FGFR1	esk, IR, ce	N/R	[235,323–325]
TP63	esk, ai, ce	ep	[105,106]
RUNX1T1	ce	N/R	[326]
PTPRC	esk	mcis	[261]
BCR	esk	N/R	[327]
BCL6	esk	N/R	[328]
ABL2	esk	N/R	[329]
BTK	esk, a3', a5'	N/R	[330]
DICER1	me	N/R	[331]
PTPN14	a5'	N/R	[332]
EPHA7	at	N/R	[333]
DAXX	a3', a5'	N/R	[334]
PRF1	sk	mcis	[104]
POLE	at	mcis	[335]
PSIP1	ce	N/R	[71,336]
PRKCD	a5'	SF	[136]
JAK2	esk	mcis	[48,337]
ABCB1	esk, ae	mcis	[338]
ABL1	esk, ce	mcis	[339]
ETV6	esk, at	mcis	[340]
MAP2K7	esk	SF	[137]
IRF1	esk, at,	mcis, SF	[138,341,342]
TGIF1	esk, ai	N/R	[343]
TNC	ce	N/R	[344]
CASZ1	esk	N/R	[345]
NSD2	esk, ai	N/R	[40]
SALL4	a5'	N/R	[346]
ETV5	ai	N/R	[347]
SMARCB1	a5'	mcis	[348]
FAS	esk	N/R	[349]
NTKR3	at	o	[350]
DHX9	esk, ce	SF, o	[142,351]
FOXO1	o	mcis	[352]
FOXP1	esk, ai	mcis, ep	[170,353]
CDK4	esk, at	mcis	[104,354,355]
DIS3	me	mcis	[356,357]
ERG	esk	N/R	[358]
NPM1	esk	N/R	[359,360]
SMC1A	esk, at, ai	SF	[143]
KLF4	esk	SF	[144]
ING1	at, ai	N/R	[361]
PTPN6	esk, ai	N/R	[362]
PRDM2	ai	ep	[171,173]
RBM39	ce	SF	[363]

CCND2	esk, at	mcis, SF, o	[114,364]
AKT3	esk, ai	SF, ep	[122,168]
NTRK1	esk	o	[365–367]
ARAF	at	SF	[145,146]
SUSD2	IR	N/R	[368]
RBFOX2	esk, a3'	N/R	[369]
CEBPA	ai	N/R	[370]
CIITA	esk	N/R	[371]
PRRX1	me	N/R	[372,373]
FGFR4	IR	N/R	[374]
RAP1GDS1	esk	SF	[147,150–152]
ZNF148	ai, ce	N/R	[375,376]
RELA	esk, a5'	N/R	[377]
NKX2-1	ai	SF	[378,379]
RBM38	esk, at	N/R	[380]
QKI	at	N/R	[381]
MDM4	IR, at	SF	[121,133,382–385]
CSF3R	esk, ce	N/R	[386]
GATA1	esk, ai	mcis	[387]
CDX2	ai, a5'	N/R	[388,389]
CHEK2	esk, a3', a5'	mcis	[219,390,391]
HGF	at	N/R	[392,393]
DAZAP1	at	o	[394]
NRP1	esk, at	N/R	[395,396]
MSI2	ai	N/R	[397]
XPC	esk, a3'	mcis, o	[398]
IKZF3	esk	N/R	[399]
BCL11A	at, ce	N/R	[400,401]
GLI1	esk, ai	N/R	[402,403]
MDM2	esk, ai, a3'	N/R	[404]
PML	esk, at	N/R	[405–407]
CBFA2T3	esk, at	ep	[174]
FANCA	esk, ce	mcis	[408,409]
NUMA1	esk, at	SF	[53,410,411]
AFF1	a3'	N/R	[412,413]
CXCR4	IR, ai	N/R	[414,415]
EWSR1	IR	SF	[148]

[1] Abbreviations used: esk, exon skipping; IR, intron retention; at, alternative termination; ai, alternative initiation; a3’ alternative 3’ splice site; a5’, alternative 5’ splice site; ce, inclusion of cryptic exon; me, mutual exclusive exons; o, other events.

[2] Molecular mechanisms involved in the splicing events. Abbreviations used: mcis, mutation in *cis* elements; ocis, other causes involving *cis* elements; SF, changes in splicing factors (mutations, change of level); ep, changes in epigenetic marks or chromatin structure; o, other causes.

SUPPLEMENTARY REFERENCES

191. Bernard, A.; Boidot, R.; Végran, F. Alternative Splicing in Cancer and Immune Cells. *Cancers* **2022**, *14*, 1726, doi:10.3390/cancers14071726.

192. Okumura, N.; Yoshida, H.; Kitagishi, Y.; Nishimura, Y.; Matsuda, S. Alternative Splicings on P53, BRCA1 and PTEN Genes Involved in Breast Cancer. *Biochemical and Biophysical Research Communications* **2011**, *413*, 395–399, doi:10.1016/j.bbrc.2011.08.098.

193. Kobayashi, Y.; Chhoeu, C.; Li, J.; Price, K.S.; Kiedrowski, L.A.; Hutchins, J.L.; Hardin, A.I.; Wei, Z.; Hong, F.; Bahcall, M.; et al. Silent Mutations Reveal Therapeutic Vulnerability in RAS Q61 Cancers. *Nature* **2022**, *603*, 335–342, doi:10.1038/s41586-022-04451-4.

194. Aran, V. K-RAS4A: Lead or Supporting Role in Cancer Biology? *Frontiers in Molecular Biosciences* **2021**, *8*, 729830, doi:10.3389/fmolb.2021.729830.

195. Salmón, M.; Paniagua, G.; Lechuga, C.G.; Fernández-García, F.; Zarzuela, E.; Álvarez-Díaz, R.; Musteanu, M.; Guerra, C.; Caleiras, E.; Muñoz, J.; et al. KRAS4A Induces Metastatic Lung Adenocarcinomas in Vivo in the Absence of the KRAS4B Isoform. *Proceedings of the National Academy of Sciences of the United States of America* **2021**, *118*, e2023112118, doi:10.1073/pnas.2023112118.

196. Marranci, A.; Jiang, Z.; Vitiello, M.; Guzzolino, E.; Comelli, L.; Sarti, S.; Lubrano, S.; Franchin, C.; Echevarría-Vargas, I.; Tuccoli, A.; et al. The Landscape of BRAF Transcript and Protein Variants in Human Cancer. *Molecular Cancer* **2017**, *16*, 85, doi:10.1186/s12943-017-0645-4.

197. Bokharaie, H.; Kolch, W.; Krstic, A. Analysis of Alternative MRNA Splicing in Vemurafenib-Resistant Melanoma Cells. *Biomolecules* **2022**, *12*, 993, doi:10.3390/biom12070993.

198. Liu, W.Q.; Dong, J.; Peng, Y.X.; Li, W.L.; Yang, J. Synonymous Mutation Adenomatous Polyposis ColiΔ486s Affects Exon Splicing and May Predispose Patients to Adenomatous Polyposis Coli/MutY DNA Glycosylase Mutation-Negative Familial Adenomatous Polyposis. *Molecular Medicine Reports* **2018**, *18*, 4931–4939, doi:10.3892/mmr.2018.9495.

199. Eisfeld, A.K.; Schwind, S.; Hoag, K.W.; Walker, C.J.; Liyanarachchi, S.; Patel, R.; Huang, X.; Markowitz, J.; Duan, W.; Otterson, G.A.; et al. NRAS Isoforms Differentially Affect Downstream Pathways, Cell Growth, and Cell Transformation. *Proceedings of the National Academy of Sciences of the United States of America* **2014**, *111*, 4179–4184, doi:10.1073/pnas.1401727111.

200. Rásó, E. Splice Variants of RAS—Translational Significance. *Cancer and Metastasis Reviews* **2020**, *39*, 1039–1049, doi:10.1007/s10555-020-09920-8.

201. Shiraishi, Y.; Kataoka, K.; Chiba, K.; Okada, A.; Kogure, Y.; Tanaka, H.; Ogawa, S.; Miyano, S. A Comprehensive Characterization of Cis-Acting Splicing-Associated Variants in Human Cancer. *Genome Research* **2018**, *28*, 1111–1125, doi:10.1101/gr.231951.117.

202. Wujcicka, W.; Zajac, A.; Szyllo, K.; Smolarz, B.; Romanowicz, H.; Stachowiak, G. Association of SNPs in CDKN2A (P14ARF) Tumour Suppressor Gene with Endometrial Cancer in Postmenopausal Women. *In Vivo* **2020**, *34*, 943–951, doi:10.21873/invivo.11862.

203. Hungate, E.A.; Vora, S.R.; Gamazon, E.R.; Moriyama, T.; Best, T.; Hulur, I.; Lee, Y.; Evans, T.J.; Ellinghaus, E.; Stanulla, M.; et al. A Variant at 9p21.3 Functionally Implicates CDKN2B in Paediatric B-Cell Precursor Acute Lymphoblastic Leukaemia Aetiology. *Nature Communications* **2016**, *7*, 10635, doi:10.1038/ncomms10635.

204. Yang, X.; Huang, W.T.; He, R.Q.; Ma, J.; Lin, P.; Xie, Z.C.; Ma, F.C.; Chen, G. Determining the Prognostic Significance of Alternative Splicing Events in Soft Tissue Sarcoma Using Data from the Cancer Genome Atlas. *Journal of Translational Medicine* **2019**, *17*, 283, doi:10.1186/s12967-019-2029-6.

205. Shah, K.; Gagliano, T.; Garland, L.; O'Hanlon, T.; Bortolotti, D.; Gentili, V.; Rizzo, R.; Giamas, G.; Dean, M. Androgen Receptor Signaling Regulates the Transcriptome of Prostate Cancer Cells by Modulating Global Alternative Splicing. *Oncogene* **2020**, *39*, 6172–6189, doi:10.1038/s41388-020-01429-2.

206. Rodríguez-Martín, C.; Cidre, F.; Fernández-Teijeiro, A.; Gómez-Mariano, G.; De La Vega, L.; Ramos, P.; Zaballos, A.; Monzón, S.; Alonso, J. Familial Retinoblastoma Due to Intronic LINE-1 Insertion Causes Aberrant and Noncanonical MRNA Splicing of the RB1 Gene. *Journal of Human Genetics* **2016**, *61*, 463–466, doi:10.1038/jhg.2015.173.

207. Yang, Z.; Wang, J.; Zhu, R. Identification of Driver Genes with Aberrantly Alternative Splicing Events in Pediatric Patients with Retinoblastoma. *Mathematical Biosciences and Engineering* **2020**, *18*, 328–338, doi:10.3934/MBE.2021017.

208. Bueno-Martínez, E.; Sanoguera-Miralles, L.; Valenzuela-Palomo, A.; Esteban-Sánchez, A.; Lorca, V.; Llinares-Burguet, I.; Allen, J.; García-Álvarez, A.; Pérez-Segura, P.; Durán, M.; et al. Minigene-Based Splicing Analysis and ACMG/AMP-Based Tentative Classification of 56 ATM Variants. *Journal of Pathology* **2022**, *258*, 83–101, doi:10.1002/path.5979.

209. Canson, D.M.; O'Mara, T.A.; Spurdle, A.B.; Glubb, D.M. Splicing Annotation of Endometrial Cancer GWAS Risk Loci Reveals Potentially Causal Variants and Supports a Role for NF1 and SKAP1 as Susceptibility Genes. *Human Genetics and Genomics Advances* **2023**, *4*, 100185, doi:10.1016/j.xhgg.2023.100185.

210. Ullah, I.; Sun, W.; Tang, L.; Feng, J. Roles of SmAds Family and Alternative Splicing Variants of Smad4 in Different Cancers. *Journal of Cancer* **2018**, *9*, 4018–4028, doi:10.7150/jca.20906.

211. de Bock, C.E.; Down, M.; Baidya, K.; Sweron, B.; Boyd, A.W.; Fiers, M.; Burns, G.F.; Molloy, T.J.; Lock, R.B.; Soulier, J.; et al. T-Cell Acute Lymphoblastic Leukemias Express a Unique Truncated FAT1 Isoform That Cooperates with NOTCH1 in Leukemia Development The. *Haematologica* **2019**, *104*, e204.

212. Yang, S.Y.; Hayer, K.E.; Fazelinia, H.; Spruce, L.A.; Asnani, M.; Black, K.L.; Naqvi, A.S.; Pillai, V.; Barash, Y.; Elenitoba-Johnson, K.S.J.; et al. FBXW7β Isoform Drives Transcriptional Activation of the Proinflammatory TNF Cluster in Human Pro-B Cells. *Blood Advances* **2023**, *7*, 1077–1091, doi:10.1182/bloodadvances.2022007910.

213. Puente, X.S.; Beà, S.; Valdés-Mas, R.; Villamor, N.; Gutiérrez-Abril, J.; Martín-Subero, J.I.; Munar, M.; Rubio-Pérez, C.; Jares, P.; Aymerich, M.; et al. Non-Coding Recurrent Mutations in Chronic Lymphocytic Leukaemia. *Nature* **2015**, *526*, 519–524, doi:10.1038/nature14666.

214. Piccione, E.C.; Lieu, T.J.; Gentile, C.F.; Williams, T.R.; Connolly, A.J.; Godwin, A.K.; Koong, A.C.; Wong, A.J. A Novel Epidermal Growth Factor Receptor Variant Lacking Multiple Domains Directly Activates Transcription and Is Overexpressed in Tumors.

Oncogene **2012**, *31*, 2953–2967, doi:10.1038/onc.2011.465.

215. Guillaudeau, A.; Durand, K.; Bessette, B.; Chaunavel, A.; Pommepuy, I.; Projetti, F.; Robert, S.; Caire, F.; Rabinovitch-Chable, H.; Labrousse, F. Egfr Soluble Isoforms and Their Transcripts Are Expressed in Meningiomas. *PLoS ONE* **2012**, *7*, e37204, doi:10.1371/journal.pone.0037204.

216. Gan, H.K.; Cvrljevic, A.N.; Johns, T.G. The Epidermal Growth Factor Receptor Variant III (EGFRvIII): Where Wild Things Are Altered. *FEBS Journal* **2013**, *280*, 5350–5370, doi:10.1111/febs.12393.

217. Wang, H.; Zhou, M.; Shi, B.; Zhang, Q.; Jiang, H.; Sun, Y.; Liu, J.; Zhou, K.; Yao, M.; Gu, J.; et al. Identification of an Exon 4-Deletion Variant of Epidermal Growth Factor Receptor with Increased Metastasis-Promoting Capacity. *Neoplasia* **2011**, *13*, 461–471, doi:10.1593/neo.101744.

218. Del Vecchio, C.A.; Giacomini, C.P.; Vogel, H.; Jensen, K.C.; Florio, T.; Merlo, A.; Pollack, J.R.; Wong, A.J. EGFRvIII Gene Rearrangement Is an Early Event in Glioblastoma Tumorigenesis and Expression Defines a Hierarchy Modulated by Epigenetic Mechanisms. *Oncogene* **2013**, *32*, 2670–2681, doi:10.1038/onc.2012.280.

219. Venkataramany, A.S.; Schieffer, K.M.; Lee, K.; Cottrell, C.E.; Wang, P.Y.; Mardis, E.R.; Cripe, T.P.; Chandler, D.S. Alternative RNA Splicing Defects in Pediatric Cancers: New Insights in Tumorigenesis and Potential Therapeutic Vulnerabilities. *Annals of Oncology* **2022**, *33*, 578–592, doi:10.1016/j.annonc.2022.03.011.

220. Beneventi, G.; Munita, R.; Cao Thi Ngoc, P.; Madej, M.; Cieśla, M.; Muthukumar, S.; Krogh, N.; Nielsen, H.; Swaminathan, V.; Bellodi, C. The Small Cajal Body-Specific RNA 15 (SCARNA15) Directs P53 and Redox Homeostasis via Selective Splicing in Cancer Cells. *NAR Cancer* **2021**, *3*, zcab026, doi:10.1093/narcan/zcab026.

221. Jayasinghe, R.G.; Cao, S.; Gao, Q.; Wendl, M.C.; Vo, N.S.; Reynolds, S.M.; Zhao, Y.; Climente-González, H.; Chai, S.; Wang, F.; et al. Systematic Analysis of Splice-Site-Creating Mutations in Cancer. *Cell Reports* **2018**, *23*, 270–281, doi:10.1016/j.celrep.2018.03.052.

222. Mallik, N.; Singh, N.; Jamwal, M.; Chhabra, S.; Hira, J.K.; Malhotra, P.; Das, R.; Sharma, P. A Novel ATRX Splice Variant Causing Acquired HbH Disease in Myelodysplastic Syndrome with Excess Blasts-1. *Cancer Genetics* **2022**, *260–261*, 53–56, doi:10.1016/j.cancergen.2021.07.001.

223. Bueno, R.; Stawiski, E.W.; Goldstein, L.D.; Durinck, S.; De Rienzo, A.; Modrusan, Z.; Gnad, F.; Nguyen, T.T.; Jaiswal, B.S.; Chirieac, L.R.; et al. Comprehensive Genomic Analysis of Malignant Pleural Mesothelioma Identifies Recurrent Mutations, Gene Fusions and Splicing Alterations. *Nature Genetics* **2016**, *48*, 407–416, doi:10.1038/ng.3520.

224. Smith, H.W.; Yang, L.; Ling, C.; Walsh, A.; Martinez, V.D.; Boucher, J.; Zuo, D.; Sokol, E.S.; Pavlick, D.C.; Frampton, G.M.; et al. An ErbB2 Splice Variant Lacking Exon 16 Drives Lung Carcinoma. *Proceedings of the National Academy of Sciences of the United States of America* **2020**, *117*, 20139–20148, doi:10.1073/PNAS.2007474117.

225. Hascoet, P.; Chesnel, F.; Jouan, F.; Le Goff, C.; Couturier, A.; Darrigrand, E.; Mahe, F.; Rioux-Leclercq, N.; Le Goff, X.; Arlot-Bonnemains, Y. The PVHL172 Isoform Is Not a Tumor Suppressor and Up-Regulates a Subset of pro-Tumorigenic Genes Including TGFB1 and MMP13. *Oncotarget* **2017**, *8*, 75989–76002, doi:10.18632/oncotarget.18376.

226. Liu, F.; Calhoun, B.; Alam, M.S.; Sun, M.; Wang, X.; Zhang, C.; Haldar, K.; Lu, X. Case Report: A Synonymous VHL Mutation (c.414A > G, p.Pro138Pro) Causes Pathogenic Familial Hemangioblastoma through Dysregulated Splicing. *BMC Medical Genetics* **2020**, *21*, 42, doi:10.1186/s12881-020-0976-7.

227. Flores, S.K.; Cheng, Z.; Jasper, A.M.; Natori, K.; Okamoto, T.; Tanabe, A.; Gotoh, K.; Shibata, H.; Sakurai, A.; Nakai, T.; et al. Synonymous but Not Silent: A Synonymous VHL Variant in Exon 2 Confers Susceptibility to Familial Pheochromocytoma and von Hippel-Lindau Disease. *Journal of Clinical Endocrinology and Metabolism* **2019**, *104*, 3826–3834, doi:10.1210/jc.2019-00235.

228. Tang, Z.; Chen, T.; Ren, X.; Zhang, Z. Identification of Transcriptional Isoforms Associated with Survival in Cancer Patient. *Journal of Genetics and Genomics* **2019**, *46*, 413–421, doi:10.1016/j.jgg.2019.08.003.

229. Qiu, L.; Wang, M.; Zhu, Y.; Xiang, Y.; Zhang, Y. A Naturally-Occurring Dominant-Negative Inhibitor of Keap1 Competitively against Its Negative Regulation of Nrf2. *International Journal of Molecular Sciences* **2018**, *19*, 2150, doi:10.3390/ijms19082150.

230. Azoitei, A.; Merseburger, A.S.; Godau, B.; Hoda, M.R.; Schmid, E.; Cronauer, M. V. C-Terminally Truncated Constitutively Active Androgen Receptor Variants and Their Biologic and Clinical Significance in Castration-Resistant Prostate Cancer. *Journal of Steroid Biochemistry and Molecular Biology* **2017**, *166*, 38–44, doi:10.1016/j.jsbmb.2016.06.008.

231. Lu, C.; Brown, L.C.; Antonarakis, E.S.; Armstrong, A.J.; Luo, J. Androgen Receptor Variant-Driven Prostate Cancer II: Advances in Laboratory Investigations. *Prostate Cancer and Prostatic Diseases* **2020**, *23*, 381–397, doi:10.1038/s41391-020-0217-3.

232. Melnyk, J.E.; Steri, V.; Nguyen, H.G.; Hann, B.; Feng, F.Y.; Shokat, K.M. The Splicing Modulator Sulfonamide Indisulam Reduces AR-V7 in Prostate Cancer Cells. *Bioorganic and Medicinal Chemistry* **2020**, *28*, 115712, doi:10.1016/j.bmc.2020.115712.

233. Kanai, M.; Göke, M.; Tsunekawa, S.; Podolsky, D.K. Signal Transduction Pathway of Human Fibroblast Growth Factor Receptor 3. Identification of a Novel 66-KDa Phosphoprotein. *Journal of Biological Chemistry* **1997**, *272*, 6621–6628, doi:10.1074/jbc.272.10.6621.

234. Tomlinson, D.C.; L'Hôte, C.G.; Kennedy, W.; Pitt, E.; Knowles, M.A. Alternative Splicing of Fibroblast Growth Factor Receptor 3 Produces a Secreted Isoform That Inhibits Fibroblast Growth Factor-Induced Proliferation and Is Repressed in Urothelial Carcinoma Cell Lines. *Cancer Research* **2005**, *65*, 10441–10449, doi:10.1158/0008-5472.CAN-05-1718.

235. Lötsch, D.; Kirchhofer, D.; Englinger, B.; Jiang, L.; Okonechnikov, K.; Senfter, D.; Laemmerer, A.; Gabler, L.; Pirker, C.; Donson, A.M.; et al. Targeting Fibroblast Growth Factor Receptors to Combat Aggressive Ependymoma. *Acta Neuropathologica* **2021**, *142*, 339–360, doi:10.1007/s00401-021-02327-x.

236. Wang, B.-D.; Lee, N.H. Aberrant RNA Splicing in Cancer and Drug Resistance. *Cancers* **2018**, *10*, 10458, doi:10.3390/cancers10110458.

237. Adamopoulos, P.G.; Tsiakanikas, P.; Boti, M.A.; Scorilas, A. Targeted Long-Read Sequencing Decodes the Transcriptional Atlas of the Founding Ras Gene Family Members. *International Journal of Molecular Sciences* **2021**, *22*, 13298, doi:10.3390/ijms222413298.

238. García-Cruz, R.; Camats, M.; Calin, G.A.; Liu, C.G.; Volinia, S.; Taccioli, C.; Croce, C.M.; Bach-Elias, M. The Role of P19 and P21 H-Ras Proteins and Mutants in MiRNA Expression in Cancer and a Costello Syndrome Cell Model. *BMC Medical Genetics* **2015**, *16*, 46, doi:10.1186/s12881-015-0184-z.

239. Thomassen, M.; Mesman, R.L.S.; Hansen, T.V.O.; Menendez, M.; Rossing, M.; Esteban-Sánchez, A.; Tudini, E.; Törngren, T.; Parsons, M.T.; Pedersen, I.S.; et al. Clinical, Splicing, and Functional Analysis to Classify BRCA2 Exon 3 Variants: Application of a Points-Based ACMG/AMP Approach. *Human Mutation* **2022**, *43*, 1921–1944, doi:10.1002/humu.24449.

240. Fraile-Bethencourt, E.; Valenzuela-Palomo, A.; Díez-Gómez, B.; Acedo, A.; Velasco, E.A. Identification of Eight Spliceogenic Variants in BRCA2 Exon 16 by Minigene Assays. *Frontiers in Genetics* **2018**, *9*, 188, doi:10.3389/fgene.2018.00188.

241. Mesman, R.L.S.; Calléja, F.M.G.R.; de la Hoya, M.; Devilee, P.; van Asperen, C.J.; Vrieling, H.; Vreeswijk, M.P.G. Alternative MRNA Splicing Can Attenuate the Pathogenicity of Presumed Loss-of-Function Variants in BRCA2. *Genetics in Medicine* **2020**, *22*, 1355–1365, doi:10.1038/s41436-020-0814-5.

242. Plotz, G.; Lopez-Garcia, L.A.; Brieger, A.; Zeuzem, S.; Biondi, R.M. Alternative AKT2 Splicing Produces Protein Lacking the Hydrophobic Motif Regulatory Region. *PLoS ONE* **2020**, *15*, e0242819, doi:10.1371/journal.pone.0242819.

243. Schmidt, J.W.; Wehde, B.L.; Sakamoto, K.; Triplett, A.A.; West, W.W.; Wagner, K.U. Novel Transcripts from a Distinct Promoter That Encode the Full-Length AKT1 in Human Breast Cancer Cells. *BMC Cancer* **2014**, *14*, 195, doi:10.1186/1471-2407-14-195.

244. Donnelly, L.L.; Hogan, T.C.; Lenahan, S.M.; Nandagopal, G.; Eaton, J.G.; Lebeau, M.A.; Mccann, C.L.; Sarausky, H.M.; Hampel, K.J.; Armstrong, J.D.; et al. Functional Assessment of Somatic STK11 Variants Identified in Primary Human Non-Small Cell Lung Cancers. *Carcinogenesis* **2021**, *42*, 1428–1438, doi:10.1093/carcin/bgab104.

245. Denison, F.C.; Hiscock, N.J.; Carling, D.; Woods, A. Characterization of an Alternative Splice Variant of LKB1. *Journal of Biological Chemistry* **2009**, *284*, 67–76, doi:10.1074/jbc.M806153200.

246. Taylor, S.E.; Martin-Hirsch, P.L.; Martin, F.L. Oestrogen Receptor Splice Variants in the Pathogenesis of Disease. *Cancer Letters* **2010**, *288*, 133–148, doi:10.1016/j.canlet.2009.06.017.

247. Ishii, H.; Hattori, Y.; Ozawa, H. Identification of a Novel C-Terminally Truncated Estrogen Receptor α Variant (ERαi34) with Constitutive Transactivation and Estrogen Receptor Antagonist Resistance. *Molecular and Cellular Endocrinology* **2020**, *503*, 110693, doi:10.1016/j.mce.2019.110693.

248. Hattori, Y.; Ishii, H.; Munetomo, A.; Watanabe, H.; Morita, A.; Sakuma, Y.; Ozawa, H. Human C-Terminally Truncated ERα Variants Resulting from the Use of Alternative Exons in the Ligand-Binding Domain. *Molecular and Cellular Endocrinology* **2016**, *425*, 111–122, doi:10.1016/j.mce.2016.01.026.

249. Dahmani, R.; Just, P.A.; Delay, A.; Canal, F.; Finzi, L.; Prip-Buus, C.; Lambert, M.; Sujobert, P.; Buchet-Poyau, K.; Miller, E.; et al. A Novel LKB1 Isoform Enhances AMPK Metabolic Activity and Displays Oncogenic Properties. *Oncogene* 2015, *34*, 2337–2346.

250. Laimer, M.; Onder, K.; Schlager, P.; Lanschuetzer, C.M.; Emberger, M.; Selhofer, S.; Hintner, H.; Bauer, J.W. Nonsense-Associated Altered Splicing of the Patched Gene Fails to Suppress Carcinogenesis in Gorlin Syndrome. *Br. J. Dermatol.* **2008**, *159*, 222–227.

251. Zhou, J.; Zhang, G.; Shi, M.; Liu, Z.; Xiao, M.; Fu, S.; Gong, X.; Shi, X. A Novel Splicing Mutation of PTCH1 in a Chinese Family with Nevoid Basal Cell Carcinoma Syndrome. *Medical Molecular Morphology* **2019**, *52*, 235–237, doi:10.1007/s00795-019-00222-9.

252. Adamia, S.; Bar-Natan, M.; Haibe-Kains, B.; Pilarski, P.M.; Bach, C.; Pevzner, S.; Calimeri, T.; Avet-Loiseau, H.; Lode, L.; Verselis, S.; et al. NOTCH2 and FLT3 Gene Mis-Splicings Are Common Events in Patients with Acute Myeloid Leukemia (AML): New Potential Targets in AML. *Blood* **2014**, *123*, 2816–2825, doi:10.1182/blood-2013-02-481507.

253. Fu, Y.P.; Edvardsen, H.; Kaushiva, A.; Arhancet, J.P.; Howe, T.M.; Kohaar, I.; Porter-Gill, P.; Shah, A.; Landmark-Høyvik, H.; Fosså, S.D.; et al. NOTCH2 in Breast Cancer: Association of SNP Rs11249433 with Gene Expression in ER-Positive Breast Tumors without TP53 Mutations. *Molecular Cancer* **2010**, *9*, 113, doi:10.1186/1476-4598-9-113.

254. Li, J.; Huang, K.; Hu, G.; Babarinde, I.A.; Li, Y.; Dong, X.; Chen, Y.S.; Shang, L.; Guo, W.; Wang, J.; et al. An Alternative CTCF Isoform Antagonizes Canonical CTCF Occupancy and Changes Chromatin Architecture to Promote Apoptosis. *Nature Communications* **2019**, *10*, doi:10.1038/s41467-019-08949-w.

255. Wan, J.; Lv, J.; Wang, C.; Zhang, L. RPS27 Selectively Regulates the Expression and Alternative Splicing of Inflammatory and Immune Response Genes in Thyroid Cancer Cells. *Advances in Clinical and Experimental Medicine* **2022**, *31*, 889–901, doi:10.17219/acem/147271.

256. Bogdanova, N. V.; Schürmann, P.; Valova, Y.; Dubrowinskaja, N.; Turmanov, N.; Yugay, T.; Essimsiitova, Z.; Mingazheva, E.; Prokofyeva, D.; Bermisheva, M.; et al. A Splice Site Variant of CDK12 and Breast Cancer in Three Eurasian Populations. *Frontiers in Oncology* **2019**, *9*, 493, doi:10.3389/fonc.2019.00493.

257. Ergün, S.; Altay, D.U.; Güneş, S.; Büyükalpelli, R.; Karahan, S.C.; Tomak, L.; Abur, Ü. Tr-KIT/c-KIT Ratio in Renal Cell Carcinoma. *Molecular Biology Reports* **2019**, *46*, 5287–5294, doi:10.1007/s11033-019-04985-3.

258. Phung, B.; Steingrímsson, E.; Rönnstrand, L. Differential Activity of C-KIT Splice Forms Is Controlled by Extracellular Peptide Insert Length. *Cellular Signalling* **2013**, *25*, 2231–2238, doi:10.1016/j.cellsig.2013.07.011.

259. Lebedev, T.D.; Vagapova, E.R.; Popenko, V.I.; Leonova, O.G.; Spirin, P. V.; Prassolov, V.S. Two Receptors, Two Isoforms, Two Cancers: Comprehensive Analysis of Kit and Trka Expression in Neuroblastoma and Acute Myeloid Leukemia. *Frontiers in Oncology* **2019**, *9*, 1046, doi:10.3389/fonc.2019.01046.

260. Jin, Y.J.; Byun, S.; Han, S.; Chamberlin, J.; Kim, D.; Kim, M.J.; Lee, Y. Differential Alternative Splicing Regulation among Hepatocellular Carcinoma with Different Risk Factors. *BMC Medical Genomics* **2019**, *12*, 175, doi:10.1186/s12920-019-0635-z.

261. Alanazi, I.O.; Alamery, S.F.; Ebrahimie, E.; Mohammadi-Dehcheshmeh, M. Splice-Disrupt Genomic Variants in Prostate Cancer. *Molecular Biology Reports* **2022**, *49*, 4237–4246, doi:10.1007/s11033-022-07257-9.

262. Karve, K.; Netherton, S.; Deng, L.; Bonni, A.; Bonni, S. Regulation of Epithelial-Mesenchymal Transition and Organoid Morphogenesis by a Novel TGFβ-TCF7L2 Isoform-Specific Signaling Pathway. *Cell Death and Disease* **2020**, *11*, 704, doi:10.1038/s41419-020-02905-z.

263. Pradas-Juni, M.; Nicod, N.; Fernández-Rebollo, E.; Gomis, R. Differential Transcriptional and Posttranslational Transcription Factor 7-like Regulation among Nondiabetic Individuals and Type 2 Diabetic Patients. *Molecular endocrinology (Baltimore, Md.)* **2014**, *28*, 1558–1570, doi:10.1210/me.2014-1065.

264. Zhang, X.; Farrell, A.S.; Daniel, C.J.; Arnold, H.; Scanlan, C.; Laraway, B.J.; Janghorban, M.; Lum, L.; Chen, D.; Troxell, M.; et al. Mechanistic Insight into Myc Stabilization in Breast Cancer Involving Aberrant Axin1 Expression. *Proc. Natl. Acad. Sci. U.S.A.* **2011**, *109*, 2790–2795, doi:10.1073/pnas.1100764108.

265. Kahles, A.; Lehmann, K. Van; Toussaint, N.C.; Hüser, M.; Stark, S.G.; Sachsenberg, T.; Stegle, O.; Kohlbacher, O.; Sander, C.; Caesar-Johnson, S.J.; et al. Comprehensive Analysis of Alternative Splicing Across Tumors from 8,705 Patients. *Cancer Cell* **2018**, *34*, 211–224, doi:10.1016/j.ccell.2018.07.001.

266. El Marabti, E.; Younis, I. The Cancer Spliceome: Reprograming of Alternative Splicing in Cancer. *Frontiers in Molecular Biosciences* **2018**, *5*, doi:10.3389/fmolb.2018.00080.

267. Li, L.; Zheng, J.; Stevens, M.; Oltean, S. A Repositioning Screen Using an FGFR2 Splicing Reporter Reveals Compounds That Regulate Epithelial-Mesenchymal Transitions and Inhibit Growth of Prostate Cancer Xenografts. *Molecular Therapy Methods and Clinical Development* **2022**, *25*, 147–157, doi:10.1016/j.omtm.2022.03.005.

268. Malhan, D.; Basti, A.; Relógio, A. Transcriptome Analysis of Clock Disrupted Cancer Cells Reveals Differential Alternative Splicing of Cancer Hallmarks Genes. *NPJ Systems Biology and Applications* **2022**, *8*, 17, doi:10.1038/s41540-022-00225-w.

269. Boti, M.A.; Adamopoulos, P.G.; Tsiakanikas, P.; Scorilas, A. Nanopore Sequencing Unveils Diverse Transcript Variants of the Epithelial Cell-Specific Transcription Factor Elf-3 in Human Malignancies. *Genes* **2021**, *12*, 839, doi:10.3390/genes12060839.

270. Lin, N.; Fu, W.; Zhao, C.; Li, B.; Yan, X.; Li, Y. Biologico-Clinical Significance of DNMT3A Variants Expression in Acute Myeloid Leukemia. *Biochemical and Biophysical Research Communications* **2017**, *494*, 270–277, doi:10.1016/j.bbrc.2017.10.041.

271. Božić, T.; Frobel, J.; Raic, A.; Ticconi, F.; Kuo, C.C.; Heilmann-Heimbach, S.; Goecke, T.W.; Zenke, M.; Jost, E.; Costa, I.G.; et al. Variants of DNMT3A Cause Transcript-Specific DNA Methylation Patterns and Affect Hematopoiesis. *Life Science Alliance* **2018**, *1*, 1–10, doi:10.26508/lsa.201800153.

272. Ishihara, T.; Hickford, D.; Fenelon, J.C.; Griffith, O.W.; Suzuki, S.; Renfree, M.B. Evolution of the Short Form of DNMT3A, DNMT3A2, Occurred in the Common Ancestor of Mammals. *Genome Biology and Evolution* **2022**, *14*, 94, doi:10.1093/gbe/evac094.

273. Cui, H.; Hu, Y.; Guo, D.; Zhang, A.; Gu, Y.; Zhang, S.; Zhao, C.; Gong, P.; Shen, X.; Li, Y.; et al. DNA Methyltransferase 3A Isoform b Contributes to Repressing E-Cadherin through Cooperation of DNA Methylation and H3K27/H3K9 Methylation in EMT-Related Metastasis of Gastric Cancer. *Oncogene* **2018**, *37*, 4358–4371, doi:10.1038/s41388-018-0285-1.

274. Zhang, Y.; Yao, D.; Zhu, X.; Zhou, J.; Ma, J.; Yang, J.; Wen, X.; Guo, H.; Lin, J.; Qian, J. DNMT3A Intragenic Hypomethylation Is Associated with Adverse Prognosis in Acute Myeloid Leukemia. *Leukemia Research* **2015**, *39*, 1041–1047, doi:10.1016/j.leukres.2015.06.015.

275. Stewart, H.J.S.; Shalit, E.; Halliday, L.; Morey, D.; Chevassut, T.J. Acute Myeloid Leukemia Cells Exhibit Selective Down-Regulation of DNMT3A Isoform 2. *Leukemia and Lymphoma* **2015**, *56*, 3445–3448, doi:10.3109/10428194.2015.1032965.

276. Warren, C.F.A.; Wong-Brown, M.W.; Bowden, N.A. BCL-2 Family Isoforms in Apoptosis and Cancer. *Cell Death and Disease* **2019**, *10*, 177, doi:10.1038/s41419-019-1407-6.

277. Lin, Y.H.; Wu, M.H.; Liu, Y.C.; Lyu, P.C.; Yeh, C.T.; Lin, K.H. LINC01348 Suppresses Hepatocellular Carcinoma Metastasis through Inhibition of SF3B3-Mediated EZH2 Pre-MRNA Splicing. *Oncogene* **2021**, *40*, 4675–4685.

278. Mu, W.; Starmer, J.; Yee, D.; Magnuson, T. EZH2 Variants Differentially Regulate Polycomb Repressive Complex 2 in Histone Methylation and Cell Differentiation. *Epigenetics and Chromatin* **2018**, *11*, 71, doi:10.1186/s13072-018-0242-9.

279. Shirahata-Adachi, M.; Iriyama, C.; Tomita, A.; Suzuki, Y.; Shimada, K.; Kiyoi, H. Altered EZH2 Splicing and Expression Is Associated with Impaired Histone H3 Lysine 27 Tri-Methylation in Myelodysplastic Syndrome. *Leukemia Research* **2017**, *63*, 90–97, doi:10.1016/j.leukres.2017.10.015.

280. Yang, Y.T.; Chiu, Y.C.; Kao, C.J.; Hou, H.A.; Lin, C.C.; Tsai, C.H.; Tseng, M.H.; Chou, W.C.; Tien, H.F. The Prognostic Significance of Global Aberrant Alternative Splicing in Patients with Myelodysplastic Syndrome. *Blood Cancer Journal* **2018**, *8*, 78, doi:10.1038/s41408-018-0115-2.

281. De Figueiredo-Pontes, L.L.; Wong, D.W.S.; Tin, V.P.C.; Chung, L.P.; Yasuda, H.; Yamaguchi, N.; Nakayama, S.; Jänne, P.A.; Wong, M.P.; Kobayashi, S.S.; et al. Identification and Characterization of ALK Kinase Splicing Isoforms in Non-Small-Cell Lung Cancer. *Journal of Thoracic Oncology* **2014**, *9*, 248–253, doi:10.1097/JTO.0000000000000050.

282. Cesi, G.; Philippidou, D.; Kozar, I.; Kim, Y.J.; Bernardin, F.; Van Niel, G.; Wienecke-Baldacchino, A.; Felten, P.; Letellier, E.; Dengler, S.; et al. A New ALK Isoform Transported by Extracellular Vesicles Confers Drug Resistance to Melanoma Cells. *Molecular Cancer* **2018**, *17*, 145, doi:10.1186/s12943-018-0886-x.

283. Seiler, M.; Yoshimi, A.; Darman, R.; Chan, B.; Keaney, G.; Thomas, M.; Agrawal, A.A.; Caleb, B.; Csibi, A.; Sean, E.; et al. H3B-8800, an Orally Available Small-Molecule Splicing Modulator, Induces Lethality in Spliceosome-Mutant Cancers. *Nature Medicine* **2018**, *24*, 497–504, doi:10.1038/nm.4493.

284. Lee, J.; Villarreal, O.D.; Chen, X.; Zandee, S.; Young, Y.K.; Torok, C.; Lamarche-Vane, N.; Prat, A.; Rivest, S.; Gosselin, D.; et al. QUAKING Regulates Microexon Alternative Splicing of the Rho GTPase Pathway and Controls Microglia Homeostasis. *Cell Reports* **2020**, *33*, 108560, doi:10.1016/j.celrep.2020.108560.

285. Wheeler, E.C.; Vora, S.; Mayer, D.; Kotini, A.G.; Olszewska, M.; Park, S.S.; Guccione, E.; Teruya-Feldstein, J.; Silverman, L.; Sunahara, R.K.; et al. Integrative RNA-Omics Discovers GNAS Alternative Splicing as a Phenotypic Driver of Splicing Factor–Mutant Neoplasms. *Cancer Discovery* **2022**, *12*, 836–855, doi:10.1158/2159-8290.CD-21-0508.

286. Spinella, J.F.; Cassart, P.; Richer, C.; Saillour, V.; Ouimet, M.; Langlois, S.; St-Onge, P.; Sontag, T.; Healy, J.; Minden, M.D.; et al. Genomic Characterization of Pediatric T-Cell Acute Lymphoblastic Leukemia Reveals Novel Recurrent Driver Mutations. *Oncotarget* **2016**, *7*, 65485–65503, doi:10.18632/oncotarget.11796.

287. Catane, L.J.; Moshel, O.; Smith, Y.; Davidson, B.; Reich, R. Splice-variant Knock-out of Tgfβ Receptors Perturbates the Proteome of Ovarian Carcinoma Cells. *International Journal of Molecular Sciences* **2021**, *22*, 12647, doi:10.3390/ijms222312647.

288. Sivadas, V.P.; Gulati, S.; Varghese, B.T.; Balan, A.; Kannan, S. The Early Manifestation, Tumor-Specific Occurrence and Prognostic Significance of TGFBR2 Aberrant Splicing in Oral Carcinoma. *Experimental Cell Research* **2014**, *327*, 156–162, doi:10.1016/j.yexcr.2014.05.004.

289. Nieke, S.; Yasmin, N.; Kakugawa, K.; Yokomizo, T.; Muroi, S.; Taniuchi, I. Unique N-Terminal Sequences in Two Runx1 Isoforms Are Dispensable for Runx1 Function. *BMC Developmental Biology* **2017**, *17*, 14, doi:10.1186/s12861-017-0156-y.

290. Martinez, M.; Hinojosa, M.; Trombly, D.; Morin, V.; Stein, J.; Stein, G.; Javed, A.; Gutierrez, S.E. Transcriptional Auto-Regulation of RUNX1 P1 Promoter. *PLoS ONE* **2016**, *11*, 149119, doi:10.1371/journal.pone.0149119.

291. Gialesaki, S.; Bräuer-Hartmann, D.; Issa, H.; Bhayadia, R.; Alejo-Valle, O.; Verboon, L.; Schmell, A.L.; Laszig, S.; Regényi, E.; Schuschel, K.; et al. RUNX1 Isoform Disequilibrium Promotes the Development of Trisomy 21–Associated Myeloid Leukemia. *Blood* **2023**, *141*, 1105–1118, doi:10.1182/blood.2022017619.

292. Genov, N.; Basti, A.; Abreu, M.; Astaburuaga, R.; Relógio, A. A Bioinformatic Analysis Identifies Circadian Expression of Splicing Factors and Time-Dependent Alternative Splicing Events in the HD-MY-Z Cell Line. *Scientific Reports* **2019**, *9*, 11062, doi:10.1038/s41598-019-47343-w.

293. Uehara, H.; Cho, Y.K.; Simonis, J.; Cahoon, J.; Archer, B.; Luo, L.; Das, S.K.; Singh, N.; Ambati, J.; Ambati, B.K. Dual Suppression of Hemangiogenesis and Lymphangiogenesis by Splice-Shifting Morpholinos Targeting Vascular Endothelial Growth Factor Receptor 2 (KDR). *FASEB Journal* **2013**, *27*, 76–85, doi:10.1096/fj.12-213835.

294. Gao, J.; Sidiropoulou, E.; Walker, I.; Krupka, J.A.; Mizielinski, K.; Usheva, Z.; Samarajiwa, S.A.; Hodson, D.J. SGK1 Mutations in DLBCL Generate Hyperstable Protein Neoisoforms That Promote AKT Independence. *Blood* **2021**, *138*, 959–964, doi:10.1182/blood.2020010432.

295. Wang, Y.; Dean, J.L.; Millar, E.K.A.; Thai, H.T.; McNeil, C.M.; Burd, C.J.; Henshall, S.M.; Utama, F.E.; Witkiewicz, A.; Rui, H.; et al. Cyclin D1b Is Aberrantly Regulated in Response to Therapeutic Challenge and Promotes Resistance to Estrogen Antagonists. *Cancer Research* **2008**, *68*, 5628–5638, doi:10.1158/0008-5472.CAN-07-3170.

296. Luo, Z.L.; Cheng, S.Q.; Shi, J.; Zhang, H.L.; Zhang, C.Z.; Chen, H.Y.; Qiu, B.J.; Tang, L.; Hu, C.L.; Wang, H.Y.; et al. A Splicing Variant of Merlin Promotes Metastasis in Hepatocellular Carcinoma. *Nature Communications* **2015**, *6*, 8457, doi:10.1038/ncomms9457.

297. Besançon, R.; Valsesia-Wittmann, S.; Locher, C.; Delloye-Bourgeois, C.; Furhman, L.; Tutrone, G.; Bertrand, C.; Jallas, A.C.; Garin, E.; Puisieux, A. Upstream ORF Affects MYCN Translation Depending on Exon 1b Alternative Splicing. *BMC Cancer* **2009**, *9*, 445, doi:10.1186/1471-2407-9-445.

298. Kaur, S.; Awad, D.; Finney, R.P.; Meyer, T.J.; Singh, S.P.; Cam, M.C.; Karim, B.O.; Warner, A.C.; Roberts, D.D. CD47-Dependent Regulation of Immune Checkpoint Gene Expression and MYCN MRNA Splicing in Murine CD8 and Jurkat T Cells. *International Journal of Molecular Sciences* **2023**, *24*, 2612, doi:10.3390/ijms24032612.

299. Fu, X.D. Both Sides of the Same Coin: Rac1 Splicing Regulating by EGF Signaling. *Cell Research* **2017**, *27*, 455–456, doi:10.1038/cr.2017.19.

300. Wang, K.; Ye, Y.; Bao, L.; Cheng, Y.; Cao, Y.; Yu, J. A Hereditary Ovarian Cancer Family with Rare Pathogenic Splicing Mutation: Implications for Variant Interpretation. *Cancer Genetics* **2021**, *256–257*, 127–130, doi:10.1016/j.cancergen.2021.05.007.

301. Ruiz de Garibay, G.; Fernandez-Garcia, I.; Mazoyer, S.; Leme de Calais, F.; Ameri, P.; Vijayakumar, S.; Martinez-Ruiz, H.; Damiola, F.; Barjhoux, L.; Thomassen, M.; et al. Altered Regulation of BRCA1 Exon 11 Splicing Is Associated with Breast Cancer Risk in Carriers of BRCA1 Pathogenic Variants. *Human Mutation* **2021**, *42*, 1488–1502, doi:10.1002/humu.24276.

302. Colombo, M.; Mondini, P.; Minenza, E.; Foglia, C.; Mosconi, A.; Molica, C.; Pistola, L.; Ludovini, V.; Radice, P. A Novel BRCA1 Splicing Variant Detected in an Early Onset Triple-Negative Breast Cancer Patient Additionally Carrying a Pathogenic Variant in ATM: A Case Report. *Frontiers in Oncology* **2023**, *13*, 1102184, doi:10.3389/fonc.2023.1102184.

303. Bhinge, K.; Yang, L.; Terra, S.; Nasir, A.; Muppa, P.; Aubry, M.C.; Yi, J.; Janaki, N.; Kovtun, I. V.; Murphy, S.J.; et al. EGFR Mediates Activation of RET in Lung Adenocarcinoma with Neuroendocrine Differentiation Characterized by ASCL1 Expression. *Oncotarget* **2017**, *8*, 27155–27165, doi:10.18632/oncotarget.15676.

304. Du, J.X.; Zhu, G.Q.; Cai, J.L.; Wang, B.; Luo, Y.H.; Chen, C.; Cai, C.Z.; Zhang, S.J.; Zhou, J.; Fan, J.; et al. Splicing Factors: Insights into Their Regulatory Network in Alternative Splicing in Cancer. *Cancer Letters* **2021**, *501*, 83–104, doi:10.1016/j.canlet.2020.11.043.

305. Tala, H.P.; Carvajal, C.A.; González, A.A.; Garrido, J.L.; Tobar, J.; Solar, A.; Campino, C.; Arteaga, E.; Fardella, C.E. New Splicing Mutation of MEN1 Gene Affecting the Translocation of Menin to the Nucleus. *J. Endocrinol. Invest.* **2006**, *29*, 888–893, doi:doi: 10.1007/BF03349192.

306. Gerber, J.M.; Gucwa, J.L.; Esopi, D.; Gurel, M.; Haffner, M.C.; Vala, M.; Nelson, W.G.; Jones, R.J.; Yegnasubramanian, S. Genome-Wide Comparison of the Transcriptomes of Highly Enriched Normal and Chronic Myeloid Leukemia Stem and Progenitor Cell Populations. *Oncotarget* **2013**, *4*, 715–728, doi:10.18632/oncotarget.990.

307. Peng, L.; Liu, Y.; Chen, J.; Cheng, M.; Wu, Y.; Chen, M.; Zhong, Y.; Shen, D.; Chen, L.; Ye, X. APEX1 Regulates Alternative Splicing of Key Tumorigenesis Genes in Non-Small-Cell Lung Cancer. *BMC Medical Genomics* **2022**, *15*, 147, doi:10.1186/s12920-022-01290-0.

308. Tolomeo, M.; Cascio, A. The Multifaced Role of Stat3 in Cancer and Its Implication for Anticancer Therapy. *International Journal of Molecular Sciences* **2021**, *22*, 603, doi:10.3390/ijms22020603.

309. Shao, H.; Quintero, A.J.; Tweardy, D.J. Identification and Characterization of Cis Elements in the STAT3 Gene Regulating STAT3α and STAT3β Messenger RNA Splicing. *Blood* **2001**, *98*, 3853–3856, doi:10.1182/blood.V98.13.3853.

310. Zammarchi, F.; De Stanchina, E.; Bournazou, E.; Supakorndej, T.; Martires, K.; Riedel, E.; Corben, A.D.; Bromberg, J.F.; Cartegni, L. Antitumorigenic Potential of STAT3 Alternative Splicing Modulation. *Proceedings of the National Academy of Sciences of the United States of America* **2011**, *108*, 17779–17784, doi:10.1073/pnas.1108482108.

311. Wali, V.B.; Haskins, J.W.; Gilmore-Hebert, M.; Platt, J.T.; Liu, Z.; Stern, D.F. Convergent and Divergent Cellular Responses by ErbB4 Isoforms in Mammary Epithelial Cells. *Molecular Cancer Research* **2014**, *12*, 1140–1155, doi:10.1158/1541-7786.MCR-13-0637.

312. Veikkolainen, V.; Vaparanta, K.; Halkilahti, K.; Iljin, K.; Sundvall, M.; Elenius, K. Function of ERBB4 Is Determined by Alternative Splicing. *Cell Cycle* **2011**, *10*, 2647–2657, doi:10.4161/cc.10.16.17194.

313. Nosi, V.; Luca, A.; Milan, M.; Arigoni, M.; Benvenuti, S.; Cacchiarelli, D.; Cesana, M.; Riccardo, S.; Filippo, L. Di; Cordero, F.; et al. Met Exon 14 Skipping: A Case Study for the Detection of Genetic Variants in Cancer Driver Genes by Deep Learning. *International Journal of Molecular Sciences* **2021**, *22*, 4217, doi:10.3390/ijms22084217.

314. Seo, J.S.; Ju, Y.S.; Lee, W.C.; Shin, J.Y.; Lee, J.K.; Bleazard, T.; Lee, J.; Jung, Y.J.; Kim, J.O.; Shin, J.Y.; et al. The Transcriptional Landscape and Mutational Profile of Lung Adenocarcinoma. *Genome Research* **2012**, *22*, 2109–2119, doi:10.1101/gr.145144.112.

315. Lee, J.; Ou, S.H.I.; Lee, J.M.; Kim, H.C.; Hong, M.; Kim, S.Y.; Jang, J.; Ahn, S.; Kang, S.Y.; Lee, S.; et al. Gastrointestinal Malignancies Harbor Actionable MET Exon 14 Deletions. *Oncotarget* **2015**, *6*, 28211–28222, doi:10.18632/oncotarget.4721.

316. Cheng, C.; Liu, L.; Bao, Y.; Yi, J.; Quan, W.; Xue, Y.; Sun, L.; Zhang, Y. SUVA: Splicing Site Usage Variation Analysis from RNA-Seq Data Reveals Highly Conserved Complex Splicing Biomarkers in Liver Cancer. *RNA Biology* **2021**, *18*, 157–171, doi:10.1080/15476286.2021.1940037.

317. Jin, Y.J.; Aycheh, H.M.; Han, S.; Chamberlin, J.; Shin, J.; Byun, S.; Lee, Y. Differential Alternative Splicing between Hepatocellular Carcinoma with Normal and Elevated Serum Alpha-Fetoprotein. *BMC Medical Genomics* **2020**, *13*, 194, doi:10.1186/s12920-020-00836-4.

318. Efthymiou, G.; Saint, A.; Ruff, M.; Rekad, Z.; Ciais, D.; Van Obberghen-Schilling, E. Shaping Up the Tumor Microenvironment With Cellular Fibronectin. *Frontiers in Oncology* **2020**, *10*, 641, doi:10.3389/fonc.2020.00641.

319. White, E.S.; Baralle, F.E.; Muro, A.F. New Insights into Form and Function of Fibronectin Splice Variants. *Journal of Pathology* **2008**, *216*, 1–14, doi:10.1002/path.2388.

320. Sun, L.; Crotty, M.L.; Sensel, M.; Sather, H.; Navara, C.; Nachman, J.; Steinherz, P.G.; Gaynon, P.S.; Seibel, N.; Mao, C.; et al. Expression of Dominant-Negative Ikaros Isoforms in T-Cell Acute Lymphoblastic Leukemia. *Clinical Cancer Research* **1999**, *5*, 2112–2120.

321. Zhao, W.; Li, Y.; Yao, C.; Zhang, G.; Zhao, K.Y.; Chen, W.; Ru, P.; Pan, X.; Tu, H.; Jones, D. Detection of Pathogenic Isoforms of IKZF1 in Leukemic Cell Lines and Acute Lymphoblastic Leukemia Samples: Identification of a Novel Truncated IKZF1 Transcript in SUP-B15. *Cancers* **2020**, *12*, 3161, doi:10.3390/cancers12113161.

322. Krishnamoorthy, G.P.; Davidson, N.R.; Leach, S.D.; Zhao, Z.; Lowe, S.W.; Lee, G.;

Landa, I.; Nagarajah, J.; Saqcena, M.; Singh, K.; et al. EIF1AX and RAS Mutations Cooperate to Drive Thyroid Tumorigenesis through ATF4 and C-MYC. *Cancer Discovery* **2019**, *9*, 264–281, doi:10.1158/2159-8290.CD-18-0606.

323. Yeh, B.K.; Igarashi, M.; Eliseenkova, A. V.; Plotnikov, A.N.; Sher, I.; Ron, D.; Aaronson, S.A.; Mohammadi, M. Structural Basis by Which Alternative Splicing Confers Specificity in Fibroblast Growth Factor Receptors. *Proceedings of the National Academy of Sciences of the United States of America* **2003**, *100*, 2266–2271, doi:10.1073/pnas.0436500100.

324. Humtsoe, J.O.; Kim, H.S.; Leonard, B.; Ling, S.; Keam, B.; Marchionni, L.; Afsari, B.; Considine, M.; Favorov, A. V.; Fertig, E.J.; et al. Newly Identified Members of FGFR1 Splice Variants Engage in Cross-Talk with AXL/AKT Axis in Salivary Adenoid Cystic Carcinoma. *Cancer Research* **2021**, *81*, 1001–1013, doi:10.1158/0008-5472.CAN-20-1780.

325. Zhao, M.; Zhuo, M.L.; Zheng, X.; Su, X.; Meric-Bernstam, F. Correction: FGFR1β Is a Driver Isoform of FGFR1 Alternative Splicing in Breast Cancer Cells (Oncotarget (2019) 10 (30-44) DOI: 10.18632/Oncotarget.26530). *Oncotarget* **2019**, *10*, 7014–7015, doi:10.18632/oncotarget.27353.

326. Ommen, H.B.; Østergaard, M.; Yan, M.; Brændstrup, K.; Zhang, D.E.; Hokland, P. Persistent Altered Fusion Transcript Splicing Identifies RUNX1-RUNX1T1+ AML Patients Likely to Relapse. *European Journal of Haematology* **2010**, *84*, 128–132, doi:10.1111/j.1600-0609.2009.01371.x.

327. Salmon, M.; White, H.E.; Zizkova, H.; Gottschalk, A.; Motlova, E.; Cerveira, N.; Colomer, D.; Coriu, D.; Franke, G.N.; Gottardi, E.; et al. Impact of BCR::ABL1 Transcript Type on RT-QPCR Amplification Performance and Molecular Response to Therapy. *Leukemia* **2022**, *36*, 1879–1886, doi:10.1038/s41375-022-01612-2.

328. Shen, Y.; Ge, B.; Ramachandrareddy, H.; McKeithan, T.; Chan, W.C. Alternative Splicing Generates a Short BCL6 (BCL6S) Isoform Encoding a Compact Repressor. *Biochemical and Biophysical Research Communications* **2008**, *375*, 190–193, doi:10.1016/j.bbrc.2008.07.116.

329. Griesinger, F.; Janke, A.; Podleschny, M.; Bohlander, S.K. Identification of an ETV6-ABL2 Fusion Transcript in Combination with an ETV6 Point Mutation in a T-Cell Acute Lymphoblastic Leukaemia Cell Line. *British Journal of Haematology* **2002**, *119*, 454–458, doi:10.1046/j.1365-2141.2002.03850.x.

330. Feldhahn, N.; Río, P.; Soh, B.N.B.; Liedtke, S.; Sprangers, M.; Klein, F.; Wernet, P.; Jumaa, H.; Hofmann, W.K.; Hanenberg, H.; et al. Deficiency of Bruton's Tyrosine Kinase in B Cell Precursor Leukemia Cells. *Proceedings of the National Academy of Sciences of the United States of America* **2005**, *102*, 13266–13271, doi:10.1073/pnas.0505196102.

331. Black, K.L.; Naqvi, A.S.; Asnani, M.; Hayer, K.E.; Yang, S.Y.; Gillespie, E.; Bagashev, A.; Pillai, V.; Tasian, S.K.; Gazzara, M.R.; et al. Aberrant Splicing in B-Cell Acute Lymphoblastic Leukemia. *Nucleic Acids Research* **2018**, *46*, 11357–11369, doi:10.1093/nar/gky946.

332. Chang, A.; Chakiryan, N.H.; Du, D.; Stewart, P.A.; Zhang, Y.; Tian, Y.; Soupir, A.C.; Bowers, K.; Fang, B.; Morganti, A.; et al. Proteogenomic, Epigenetic, and Clinical Implications of Recurrent Aberrant Splice Variants in Clear Cell Renal Cell Carcinoma. *European Urology* **2022**, *82*, 354–362, doi:10.1016/j.eururo.2022.05.021.

333. Oricchio, E.; Nanjangud, G.; Wolfe, A.L.; Schatz, J.H.; Mavrakis, K.J.; Jiang, M.; Liu, X.; Bruno, J.; Heguy, A.; Olshen, A.B.; et al. The Eph-Receptor A7 Is a Soluble Tumor Suppressor for Follicular Lymphoma. *Cell* **2011**, *147*, 554–564, doi:10.1016/j.cell.2011.09.035.

334. Wethkamp, N.; Hanenberg, H.; Funke, S.; Suschek, C. V.; Wetzel, W.; Heikaus, S.; Grinstein, E.; Ramp, U.; Engers, R.; Gabbert, H.E.; et al. Daxx-β and Daxx-γ, Two Novel Splice Variants of the Transcriptional Co-Repressor Daxx. *Journal of Biological Chemistry* **2011**, *286*, 19576–19588, doi:10.1074/jbc.M110.196311.

335. Steiner, M.; Gassner, F.J.; Parigger, T.; Neureiter, D.; Egle, A.; Geisberger, R.; Greil, R.; Zaborsky, N. A POLE Splice Site Deletion Detected in a Patient with Biclonal CLL and Prostate Cancer: A Case Report. *International Journal of Molecular Sciences* **2021**, *22*, 9410, doi:10.3390/ijms22179410.

336. Singh, D.P.; Kimura, A.; Chylack, L.T.; Shinohara, T. Lens Epithelium-Derived Growth Factor (LEDGF/P75) and P52 Are Derived from a Single Gene by Alternative Splicing. *Gene* **2000**, *242*, 265–273, doi:10.1016/S0378-1119(99)00506-5.

337. Ma, W.; Kantarjian, H.; Zhang, X.; Yen, C.-H.; Zhang, Z.J.; Verstovsek, S.; Albitar, M. Mutation Profile of JAK2 Transcripts in Patients with Chronic Myeloproliferative Neoplasias. *Journal of Molecular Diagnostics* **2009**, *11*, 49–53, doi:10.2353/jmoldx.2009.080114.

338. Li, M.; Kong, X.Y.; Wang, S.M. Effects of Splicing-Regulatory Polymorphisms in ABCC2, ABCG2, and ABCB1 on Methotrexate Exposure in Chinese Children with Acute Lymphoblastic Leukemia. *Cancer Chemotherapy and Pharmacology* **2023**, *91*, 77–87, doi:10.1007/s00280-022-04498-0.

339. Cavelier, L.; Ameur, A.; Häggqvist, S.; Höijer, I.; Cahill, N.; Olsson-Strömberg, U.; Hermanson, M. Clonal Distribution of BCR-ABL1 Mutations and Splice Isoforms by Single-Molecule Long-Read RNA Sequencing. *BMC Cancer* **2015**, *15*, 45, doi:10.1186/s12885-015-1046-y.

340. Noetzli, L.; Lo, R.W.; Lee-Sherick, A.B.; Callaghan, M.; Noris, P.; Savoia, A.; Rajpurkar, M.; Jones, K.; Gowan, K.; Balduini, C.L.; et al. Germline Mutations in ETV6 Are Associated with Thrombocytopenia, Red Cell Macrocytosis and Predisposition to Lymphoblastic Leukemia. *Nature Genetics* **2015**, *47*, 535–538, doi:10.1038/ng.3253.

341. Lee, E.J.; Jo, M.; Park, J.; Zhang, W.; Lee, J.H. Alternative Splicing Variants of IRF-1 Lacking Exons 7, 8, and 9 in Cervical Cancer. *Biochemical and Biophysical Research Communications* **2006**, *347*, 882–888, doi:10.1016/j.bbrc.2006.06.145.

342. Maratheftis, C.I.; Bolaraki, P.E.; Giannouli, S.; Kapsogeorgou, E.K.; Moutsopoulos, H.M.; Voulgarelis, M. Aberrant Alternative Splicing of Interferon Regulatory Factor-1 (IRF-1) in Myelodysplastic Hematopoietic Progenitor Cells. *Leukemia Research* **2006**, *30*, 1177–1186, doi:10.1016/j.leukres.2005.12.021.

343. Libório, T.N.; Ferreira, E.N.; Aquino Xavier, F.C.; Carraro, D.M.; Kowalski, L.P.; Soares, F.A.; Nunes, F.D. TGIF1 Splicing Variant 8 Is Overexpressed in Oral Squamous Cell Carcinoma and Is Related to Pathologic and Clinical Behavior. *Oral Surgery, Oral Medicine, Oral Pathology and Oral Radiology* **2013**, *116*, 614–625, doi:10.1016/j.oooo.2013.07.014.

344. Tsunoda, T.; Inada, H.; Kalembeyi, I.; Imanaka-Yoshida, K.; Sakakibara, M.; Okada, R.; Katsuta, K.; Sakakura, T.; Majima, Y.; Yoshida, T. Involvement of Large Tenascin-C Splice Variants in Breast Cancer Progression. *American Journal of Pathology* **2003**, *162*,

1857–1867, doi:10.1016/S0002-9440(10)64320-9.

345. Wu, Y.Y.; Chang, C.L.; Chuang, Y.J.; Wu, J.E.; Tung, C.H.; Chen, Y.C.; Chen, Y.L.; Hong, T.M.; Hsu, K.F. CASZ1 Is a Novel Promoter of Metastasis in Ovarian Cancer. *American Journal of Cancer Research* **2016**, *6*, 1253–1270.

346. Ma, Y.; Cui, W.; Yang, J.; Qu, J.; Di, C.; Amin, H.M.; Lai, R.; Ritz, J.; Krause, D.S.; Chai, L. SALL4, a Novel Oncogene, Is Constitutively Expressed in Human Acute Myeloid Leukemia (AML) and Induces AML in Transgenic Mice. *Blood* **2006**, *108*, 2726–2735, doi:10.1182/blood-2006-02-001594.

347. Alagaratnam, S..; Harrison, N.; Bakken, A.C.; Hoff, A.M.; Jones, M.; Sveen, A.; Moore, H.D.; Andrews, P.W.; Lothe, R.A.; Skotheim, R.I. Transforming Pluripotency: An Exon-Level Study of Malignancy-Specific Transcripts in Human Embryonal Carcinoma and Embryonic Stem Cells. *Stem Cells Dev.* **2013**, *22*, 1136–1146, doi:10.1089/scd.2012.0369.

348. Melean, G.; Velasco, A.; Hernández-Imaz, E.; Rodríguez-Álvarez, F.J.; Martín, Y.; Valero, A.; Hernández-Chico, C. RNA-Based Analysis of Two SMARCB1 Mutations Associated with Familial Schwannomatosis with Meningiomas. *Neurogenetics* **2012**, *13*, 267–274, doi:10.1007/s10048-012-0335-8.

349. Choi, N.; Jang, H.N.; Oh, J.; Ha, J.; Park, H.; Zheng, X.; Lee, S.; Shen, H. SRSF6 Regulates the Alternative Splicing of the Apoptotic Fas Gene by Targeting a Novel RNA Sequence. *Cancers* **2022**, *14*, 1990, doi:10.3390/cancers14081990.

350. Guidi, M.; Muiños-Gimeno, M.; Kagerbauer, B.; Martí, E.; Estivill, X.; Espinosa-Parrilla, Y. Overexpression of MiR-128 Specifically Inhibits the Truncated Isoform of NTRK3 and Upregulates BCL2 in SH-SY5Y Neuroblastoma Cells. *BMC Molecular Biology* **2010**, *11*, 95, doi:10.1186/1471-2199-11-95.

351. Fidaleo, M.; Svetoni, F.; Volpe, E.; Miñana, B.; Caporossi, D.; Paronetto, M.P. Genotoxic Stress Inhibits Ewing Sarcoma Cell Growth by Modulating Alternative Pre-MRNA Processing of the RNA Helicase DHX9. *Oncotarget* **2015**, *6*, 31740–31757, doi:10.18632/oncotarget.5033.

352. Dallas, P.B.; Egli, S.; Terry, P.A.; Kees, U.R. Aberrant Over-Expression of a Forkhead Family Member, FOXO1A, in a Brain Tumor Cell Line. *BMC Cancer* **2007**, *7*, 67, doi:10.1186/1471-2407-7-67.

353. van Keimpema, M.; Grüneberg, L.J.; Schilder-Tol, E.J.M.; Oud, M.E.C.M.; Beuling, E.A.; Hensbergen, P.J.; de Jong, J.; Pals, S.T.; Spaargaren, M. The Small FOXP1 Isoform Predominantly Expressed in Activated B Cell-like Diffuse Large B-Cell Lymphoma and Full-Length FOXP1 Exert Similar Oncogenic and Transcriptional Activity in Human B Cells. *Haematologica* **2017**, *102*, 573–583, doi:10.3324/haematol.2016.156455.

354. Adamopoulos, P.G.; Athanasopoulou, K.; Tsiakanikas, P.; Scorilas, A. A Comprehensive Nanopore Sequencing Methodology Deciphers the Complete Transcriptional Landscape of Cyclin-Dependent Kinase 4 (CDK4) in Human Malignancies. *FEBS Journal* **2022**, *289*, 712–729, doi:10.1111/febs.16201.

355. Wafa, K.; MacLean, J.; Zhang, F.; Pasumarthi, K.B.S. Characterization of Growth Suppressive Functions of a Splice Variant of Cyclin D2. *PLoS ONE* **2013**, *8*, e53503, doi:10.1371/journal.pone.0053503.

356. Robinson, S.R.; Viegas, S.C.; Matos, R.G.; Domingues, S.; Bedir, M.; Stewart, H.J.S.;

Chevassut, T.J.; Oliver, A.W.; Arraiano, C.M.; Newbury, S.F. Dis3 Isoforms Vary in Their Endoribonuclease Activity and Are Differentially Expressed within Haematological Cancers. *Biochemical Journal* **2018**, *475*, 2091–2105, doi:10.1042/BCJ20170962.

357. Liu, E.; Becker, N.; Sudha, P.; Dong, C.; Liu, Y.; Keats, J.; Morgan, G.; Walker, B.A. Alternative Splicing in Multiple Myeloma Is Associated with the Non-Homologous End Joining Pathway. *Blood Cancer Journal* **2023**, *13*, 16, doi:10.1038/s41408-023-00783-0.

358. Hagen, R.M.; Adamo, P.; Karamat, S.; Oxley, J.; Aning, J.J.; Gillatt, D.; Persad, R.; Ladomery, M.R.; Rhodes, A. Quantitative Analysis of ERG Expression and Its Splice Isoforms in Formalin-Fixed, Paraffin-Embedded Prostate Cancer Samples: Association with Seminal Vesicle Invasion and Biochemical Recurrence. *American Journal of Clinical Pathology* **2014**, *142*, 533–540, doi:10.1309/AJCPH88QHXARISUP.

359. Szelest, M.; Masternak, M.; Zając, M.; Chojnacki, M.; Skórka, K.; Zaleska, J.; Karczmarczyk, A.; Stasiak, G.; Wawrzyniak, E.; Kotkowska, A.; et al. The Role of NPM1 Alternative Splicing in Patients with Chronic Lymphocytic Leukemia. *PLoS ONE* **2022**, *17*, 276674, doi:10.1371/journal.pone.0276674.

360. Handschuh, L.; Wojciechowski, P.; Kazmierczak, M.; Marcinkowska-Swojak, M.; Luczak, M.; Lewandowski, K.; Komarnicki, M.; Blazewicz, J.; Figlerowicz, M.; Kozlowski, P. NPM1 Alternative Transcripts Are Upregulated in Acute Myeloid and Lymphoblastic Leukemia and Their Expression Level Affects Patient Outcome. *Journal of Translational Medicine* **2018**, *16*, 232, doi:10.1186/s12967-018-1608-2.

361. Melekhova, A.; Baniahmad, A. Ing Tumour Suppressors and Ing Splice Variants as Coregulators of the Androgen Receptor Signalling in Prostate Cancer. *Cells* **2021**, *10*, 2599, doi:10.3390/cells10102599.

362. Evren, S.; Wan, S.; Ma, X.Z.; Fahim, S.; Mody, N.; Sakac, D.; Jin, T.; Branch, D.R. Characterization of SHP-1 Protein Tyrosine Phosphatase Transcripts, Protein Isoforms and Phosphatase Activity in Epithelial Cancer Cells. *Genomics* **2013**, *102*, 491–499, doi:10.1016/j.ygeno.2013.10.001.

363. Campagne, S.; Jutzi, D.; Malard, F.; Matoga, M.; Romane, K.; Feldmuller, M.; Colombo, M.; Ruepp, M.D.; Allain, F.H.T. Molecular Basis of RNA-Binding and Autoregulation by the Cancer-Associated Splicing Factor RBM39. *Nature Communications* **2023**, *14*, 5366, doi:10.1038/s41467-023-40254-5.

364. Misiewicz-Krzeminska, I.; Sarasquete, M.E.; Vicente-Dueñas, C.; Krzeminski, P.; Wiktorska, K.; Corchete, L.A.; Quwaider, D.; Rojas, E.A.; Corral, R.; Martín, A.A.; et al. Post-Transcriptional Modifications Contribute to the Upregulation of Cyclin D2 in Multiple Myeloma. *Clinical Cancer Research* **2016**, *22*, 207–217, doi:10.1158/1078-0432.CCR-14-2796.

365. Tacconelli, A.; Farina, A.R.; Cappabianca, L.; DeSantis, G.; Tessitore, A.; Vetuschi, A.; Sferra, R.; Rucci, N.; Argenti, B.; Screpanti, I.; et al. TrkA Alternative Splicing: A Regulated Tumor-Promoting Switch in Human Neuroblastoma. *Cancer Cell* **2004**, *6*, 347–360, doi:10.1016/j.ccr.2004.09.011.

366. Cappabianca, L.; Zelli, V.; Pellegrini, C.; Sebastiano, M.; Maccarone, R.; Clementi, M.; Chiominto, A.; Ruggeri, P.; Cardelli, L.; Ruggieri, M.; et al. The Alternative TrkAIII Splice Variant, a Targetable Oncogenic Participant in Human Cutaneous Malignant Melanoma. *Cells* **2023**, *12*, 237, doi:10.3390/cells12020237.

367. Cappabianca, L.; Guadagni, S.; Maccarone, R.; Sebastiano, M.; Chiominto, A.; Farina,

A.R.; Mackay, A.R. A Pilot Study of Alternative TrkAIII Splicing in Merkel Cell Carcinoma: A Potential Oncogenic Mechanism and Novel Therapeutic Target. *Journal of Experimental and Clinical Cancer Research* **2019**, *38*, 424, doi:10.1186/s13046-019-1425-3.

368. Pio, R.; Blanco, D.; Pajares, M.J.; Aibar, E.; Durany, O.; Ezponda, T.; Agorreta, J.; Gomez-Roman, J.; Anton, M.A.; Rubio, A.; et al. Development of a Novel Splice Array Platform and Its Application in the Identification of Alternative Splice Variants in Lung Cancer. *BMC Genomics* **2010**, *11*, 352, doi:10.1186/1471-2164-11-352.

369. Choi, S.; Cho, N.; Kim, K.K. Non-Canonical Splice Junction Processing Increases the Diversity of RBFOX2 Splicing Isoforms. *International Journal of Biochemistry and Cell Biology* **2022**, *144*, 106172, doi:10.1016/j.biocel.2022.106172.

370. Garcia-Cuellar, M.P.; Akan, S.; Slany, R.K. A C/Ebpα Isoform Specific Differentiation Program in Immortalized Myelocytes. *Leukemia* **2023**, *37*, 1850–1859, doi:10.1038/s41375-023-01989-8.

371. Chiu, B.L.; Li, C.H.; Chang, C.C. Selective Modulation of MHC Class II Chaperons by a Novel IFN-γ-Inducible Class II Transactivator Variant in Lung Adenocarcinoma A549 Cells. *Biochemical and Biophysical Research Communications* **2013**, *440*, 190–195, doi:10.1016/j.bbrc.2013.09.066.

372. Marchand, B.; Pitarresi, J.R.; Reichert, M.; Suzuki, K.; Laczkó, D.; Rustgi, A.K. PRRX1 Isoforms Cooperate with FOXM1 to Regulate the DNA Damage Response in Pancreatic Cancer Cells. *Oncogene* **2019**, *38*, 4325–4339, doi:10.1038/s41388-019-0725-6.

373. Sun, L.; Han, T.; Zhang, X.; Liu, X.; Li, P.; Shao, M.; Dong, S.; Li, W. PRRX1 Isoform PRRX1A Regulates the Stemness Phenotype and Epithelial-Mesenchymal Transition (EMT) of Cancer Stem-like Cells (CSCs) Derived from Non-Small Cell Lung Cancer (NSCLC). *Translational Lung Cancer Research* **2020**, *9*, 731–744, doi:10.21037/tlcr-20-633.

374. Takaishi, S.; Sawada, M.; Morita, Y.; Seno, H.; Fukuzawa, H.; Chiba, T. Identification of a Novel Alternative Splicing of Human FGF Receptor 4: Soluble-Form Splice Variant Expressed in Human Gastrointestinal Epithelial Cells. *Biochemical and Biophysical Research Communications* **2000**, *267*, 658–662, doi:10.1006/bbrc.1999.2010.

375. Law, D.J.; Labut, E.M.; Adams, R.D.; Merchant, J.L. An Isoform of ZBP-89 Predisposes the Colon to Colitis. *Nucleic Acids Research* **2006**, *34*, 1342–1350, doi:10.1093/nar/gkl022.

376. Liu, Y.; Huang, W.; Gao, X.; Kuang, F. Regulation between Two Alternative Splicing Isoforms ZNF148 FL and ZNF148 ΔN , and Their Roles in the Apoptosis and Invasion of Colorectal Cancer. *Pathology Research and Practice* **2019**, *215*, 272–277, doi:10.1016/j.prp.2018.10.036.

377. Xargay-Torrent, S.; López-Guerra, M.; Rosich, L.; Montraveta, A.; Roldán, J.; Rodríguez, V.; Villamor, N.; Aymerich, M.; Lagisetti, C.; Webb, T.R.; et al. The Splicing Modulator Sudemycin Induces a Specific Antitumor Response and Cooperates with Ibrutinib in Chronic Lymphocytic Leukemia. *Oncotarget* **2015**, *6*, 22734–22749, doi:10.18632/oncotarget.4212.

378. Yamaguchi, T.; Hosono, Y.; Yanagisawa, K.; Takahashi, T. NKX2-1/TTF-1: An Enigmatic Oncogene That Functions as a Double-Edged Sword for Cancer Cell Survival and Progression. *Cancer Cell* **2013**, *23*, 718–723, doi:10.1016/j.ccr.2013.04.002.

379. Sano, K.; Hayashi, T.; Suehara, Y.; Hosoya, M.; Takamochi, K.; Kohsaka, S.; Kishikawa, S.; Kishi, M.; Saito, S.; Takahashi, F.; et al. Transcription Start Site-Level Expression of Thyroid Transcription Factor 1 Isoforms in Lung Adenocarcinoma and Its Clinicopathological Significance. *Journal of Pathology: Clinical Research* **2021**, *7*, 361–374, doi:10.1002/cjp2.213.

380. Shu, L.; Yan, W.; Chen, X. RNPC1, an RNA-Binding Protein and a Target of the P53 Family, Is Required for Maintaining the Stability of the Basal and Stress-Induced P21 Transcript. *Genes & development* **2006**, *20*, 2961–2972, doi:10.1101/gad.1463306.

381. Pilotte, J.; Larocque, D.; Richard, S. Nuclear Translocation Controlled by Alternatively Spliced Isoforms Inactivates the QUAKING Apoptotic Inducer. *Genes and Development* **2001**, *15*, 845–858, doi:10.1101/gad.860301.

382. Rallapalli, R.; Strachan, G.; Cho, B.; Mercer, W.E.; Hall, D.J. A Novel MDMX Transcript Expressed in a Variety of Transformed Cell Lines Encodes a Truncated Protein with Potent P53 Repressive Activity. *Journal of Biological Chemistry* **1999**, *274*, 8299–8308, doi:10.1074/jbc.274.12.8299.

383. Mancini, F.; Conza, G.; Moretti, F. MDM4 (MDMX) and Its Transcript Variants. *Current Genomics* **2009**, *10*, 42–50, doi:10.2174/138920209787581280.

384. Bielli, P.; Pagliarini, V.; Pieraccioli, M.; Caggiano, C.; Sette, C. Splicing Dysregulation as Oncogenic Driver and Passenger Factor in Brain Tumors. *Cells* **2020**, *9*, 10, doi:10.3390/cells9010010.

385. Pavlyukov, M.S.; Yu, H.; Bastola, S.; Minata, M.; Shender, V.O.; Lee, Y.; Zhang, S.; Wang, J.; Komarova, S.; Wang, J.; et al. Apoptotic Cell-Derived Extracellular Vesicles Promote Malignancy of Glioblastoma Via Intercellular Transfer of Splicing Factors. *Cancer Cell* **2018**, *34*, 119–135, doi:10.1016/j.ccell.2018.05.012.

386. Mehta, H.M.; Futami, M.; Glaubach, T.; Lee, D.W.; Andolina, J.R.; Yang, Q.; Whichard, Z.; Quinn, M.; Lu, H.F.; Kao, W.M.; et al. Alternatively Spliced, Truncated GCSF Receptor Promotes Leukemogenic Properties and Sensitivity to JAK Inhibition. *Leukemia* **2014**, *28*, 1041–1051, doi:10.1038/leu.2013.321.

387. Zucker, J.; Temm, C.; Czader, M.; Nalepa, G. A Child With Dyserythropoietic Anemia and Megakaryocyte Dysplasia Due to a Novel 5′UTR GATA1s Splice Mutation. *Pediatric Blood and Cancer* **2016**, *63*, 917–921, doi:10.1002/pbc.25871.

388. Witek, M.E.; Snook, A.E.; Lin, J.E.; Blomain, E.S.; Xiang, B.; Magee, M.; Waldman, S.A. A Novel CDX2 Isoform Regulates Alternative Splicing. *PLoS ONE* **2014**, *9*, 04293, doi:10.1371/journal.pone.0104293.

389. Balbinot, C.; Vanier, M.; Armant, O.; Nair, A.; Penichon, J.; Soret, C.; Martin, E.; Saandi, T.; Reimund, J.M.; Deschamps, J.; et al. Fine-Tuning and Autoregulation of the Intestinal Determinant and Tumor Suppressor Homeobox Gene CDX2 by Alternative Splicing. *Cell Death and Differentiation* **2017**, *24*, 2173–2186, doi:10.1038/cdd.2017.140.

390. Staalesen, V.; Falck, J.; Geisler, S.; Bartkova, J.; Børresen-Dale, A.L.; Lukas, J.; Lillehaug, J.R.; Bartek, J.; Lønning, P.E. Alternative Splicing and Mutation Status of CHEK2 in Stage III Breast Cancer. *Oncogene* **2004**, *23*, 8535–8544, doi:10.1038/sj.onc.1207928.

391. Berge, E.O.; Staalesen, V.; Straume, A.H.; Lillehaug, J.R.; Lønning, P.E. Chk2 Splice Variants Express a Dominant-Negative Effect on the Wild-Type Chk2 Kinase Activity.

Biochimica et Biophysica Acta - Molecular Cell Research **2010**, *1803*, 386–395, doi:10.1016/j.bbamcr.2010.01.005.

392. Frisch, R.N.; Curtis, K.M.; Aenlle, K.K.; Howard, G.A. Hepatocyte Growth Factor and Alternative Splice Variants - Expression, Regulation and Implications in Osteogenesis and Bone Health and Repair. *Expert Opinion on Therapeutic Targets* **2016**, *20*, 1087–1098, doi:10.1517/14728222.2016.1162293.

393. Mungunsukh, O.; Lee, Y.H.; Bottaro, D.P.; Day, R.M. The Hepatocyte Growth Factor Isoform NK2 Activates Motogenesis and Survival but Not Proliferation Due to Lack of Akt Activation. *Cellular Signalling* **2016**, *28*, 1114–1123, doi:10.1016/j.cellsig.2016.05.012.

394. Yang, C.K.; Yen, P. Differential Translation of Dazap1 Transcripts during Spermatogenesis. *PLoS ONE* **2013**, *8*, 60873, doi:10.1371/journal.pone.0060873.

395. Rossignol, M.; Gagnon, M.L.; Klagsbrun, M. Genomic Organization of Human Neuropilin-1 and Neuropilin-2 Genes: Identification and Distribution of Splice Variants and Soluble Isoforms. *Genomics* **2000**, *70*, 211–222, doi:10.1006/geno.2000.6381.

396. Cackowski, F.C.; Xu, L.; Hu, B.; Cheng, S.Y. Identification of Two Novel Alternatively Spliced Neuropilin-1 Isoforms. *Genomics* **2004**, *84*, 82–94, doi:10.1016/j.ygeno.2004.02.001.

397. Mansouri, L.; Gunnarsson, R.; Sutton, L.A.; Ameur, A.; Hooper, S.D.; Mayrhofer, M.; Juliusson, G.; Isaksson, A.; Gyllensten, U.; Rosenquist, R. Next Generation RNA-Sequencing in Prognostic Subsets of Chronic Lymphocytic Leukemia. *American Journal of Hematology* **2012**, *87*, 737–740, doi:10.1002/ajh.23227.

398. Cartault, F.; Nava, C.; Malbrunot, A.C.; Munier, P.; Hebert, J.C.; N'guyen, P.; Djeridi, N.; Pariaud, P.; Pariaud, J.; Dupuy, A.; et al. A New XPC Gene Splicing Mutation Has Lead to the Highest Worldwide Prevalence of Xeroderma Pigmentosum in Black Mahori Patients. *DNA Repair* **2011**, *10*, 577–585, doi:10.1016/j.dnarep.2011.03.005.

399. Caballero, R.; Setien, F.; Lopez-Serra, L.; Boix-Chornet, M.; Fraga, M.F.; Ropero, S.; Megias, D.; Alaminos, M.; Sanchez-Tapia, E.M.; Montoya, M.C.; et al. Combinatorial Effects of Splice Variants Modulate Function of Aiolos. *Journal of Cell Science* **2007**, *120*, 2619–2630, doi:10.1242/jcs.007344.

400. Liu, H.; Ippolito, G.C.; Wall, J.K.; Niu, T.; Probst, L.; Lee, B.S.; Pulford, K.; Banham, A.H.; Stockwin, L.; Shaffer, A.L.; et al. Functional Studies of BCLIIA: Characterization of the Conserved BCLIIA-XL Splice Variant and Its Interaction with BCL6 in Nuclear Paraspeckles of Germinal Center B Cells. *Molecular Cancer* **2006**, *5*, 18, doi:10.1186/1476-4598-5-18.

401. Zhang, N.; Jiang, B.Y.; Zhang, X.C.; Xie, Z.; Su, J.; Zhang, Q.; Han, J.F.; Tu, H.Y.; Wu, Y.L. The BCL11A-XL Expression Predicts Relapse in Squamous Cell Carcinoma and Large Cell Carcinoma. *Journal of Thoracic Disease* **2015**, *7*, 1630–1636, doi:10.3978/j.issn.2072-1439.2015.09.39.

402. Lo, H.W.; Zhu, H.; Cao, X.; Aldrich, A.; Ali-Osman, F. A Novel Splice Variant of GLI1 That Promotes Glioblastoma Cell Migration and Invasion. *Cancer Research* **2009**, *69*, 6790–6798, doi:10.1158/0008-5472.CAN-09-0886.

403. Shimokawa, T.; Tostar, U.; Lauth, M.; Palaniswamy, R.; Kasper, M.; Toftgård, R.; Zaphiropoulos, P.G. Novel Human Glioma-Associated Oncogene 1 (GLI1) Splice Variants Reveal Distinct Mechanisms in the Terminal Transduction of the Hedgehog

Signal. *Journal of Biological Chemistry* **2008**, *283*, 14345–14354, doi:10.1074/jbc.M800299200.

404. Bartel, F.; Taubert, H.; Harris, L.C. Alternative and Aberrant Splicing of MDM2 MRNA in Human Cancer. *Cancer Cell* **2002**, *2*, 9–15, doi:10.1016/S1535-6108(02)00091-0.

405. Zhang, Y.; Shao, A. wen; Tang, J.; Geng, Y. PML-II Recruits Ataxin-3 to PML-NBs and Inhibits Its Deubiquitinating Activity. *Biochemical and Biophysical Research Communications* **2021**, *554*, 186–192, doi:10.1016/j.bbrc.2021.03.098.

406. Lim, J.; Choi, J.H.; Park, E.M.; Choi, Y.H. Interaction of Promyelocytic Leukemia/P53 Affects Signal Transducer and Activator of Transcription-3 Activity in Response to Oncostatin M. *Korean Journal of Physiology and Pharmacology* **2020**, *24*, 203–212, doi:10.4196/KJPP.2020.24.3.203.

407. El-Asmi, F.; Chelbi-Alix, M.K. Les Isoformes de PML et La Réponse Au TGF-β. *Médecine/Sciences* **2020**, *36*, 50–56, doi:10.1051/medsci/2019269.

408. Litim, N.; Labrie, Y.; Desjardins, S.; Ouellette, G.; Plourde, K.; Belleau, P.; Durocher, F. Polymorphic Variations in the FANCA Gene in High-Risk Non-BRCA1/2 Breast Cancer Individuals from the French Canadian Population. *Molecular Oncology* **2013**, *7*, 85–100, doi:10.1016/j.molonc.2012.08.002.

409. Mattioli, C.; Pianigiani, G.; De Rocco, D.; Bianco, A.M.R.; Cappelli, E.; Savoia, A.; Pagani, F. Unusual Splice Site Mutations Disrupt FANCA Exon 8 Definition. *Biochimica et Biophysica Acta - Molecular Basis of Disease* **2014**, *1842*, 1052–1058, doi:10.1016/j.bbadis.2014.03.014.

410. Park, J.; Kim, D.; Lee, J.O.; Park, H.C.; Ryu, B.Y.; Kim, J.H.; Lee, S.H.; Chung, Y.J. Dissection of Molecular and Histological Subtypes of Papillary Thyroid Cancer Using Alternative Splicing Profiles. *Experimental and Molecular Medicine* **2022**, *54*, 263–272, doi:10.1038/s12276-022-00740-0.

411. Wu, J.; Xu, Z.; He, D.; Lu, G. Identification and Characterization of Novel NuMA Isoforms. *Biochemical and Biophysical Research Communications* **2014**, *454*, 387–392, doi:10.1016/j.bbrc.2014.10.104.

412. Janssen, J.W.G.; Ludwig, W.D.; Borkhardt, A.; Spadinger, U.; Rieder, H.; Fonatsch, C.; Hossfeld, D.K.; Harbott, J.; Schulz, A.S.; Repp, R.; et al. Pre-Pre-B Acute Lymphoblastic Leukemia: High Frequency of Alternatively Spliced ALL1-AF4 Transcripts and Absence of Minimal Residual Disease during Complete Remission. *Blood* **1994**, *84*, 3835–3842, doi:10.1182/blood.v84.11.3835.bloodjournal84113835.

413. Ragusa, D.; Makarov, E.M.; Britten, O.; Moralli, D.; Green, C.M.; Tosi, S. The RS4;11 Cell Line as a Model for Leukaemia with t(4;11)(Q21;Q23): Revised Characterisation of Cytogenetic Features. *Cancer Reports* **2019**, *2*, e1207, doi:10.1002/cnr2.1207.

414. Gupta, S.K.; Pillarisetti, K. Cutting Edge: CXCR4-Lo: Molecular Cloning and Functional Expression of a Novel Human CXCR4 Splice Variant. *The Journal of Immunology* **1999**, *163*, 2368–2372, doi:10.4049/jimmunol.163.5.2368.

415. Sand, L.G.L.; Jochemsen, A.G.; Beletkaia, E.; Schmidt, T.; Hogendoorn, P.C.W.; Szuhai, K. Novel Splice Variants of CXCR4 Identified by Transcriptome Sequencing. *Biochemical and Biophysical Research Communications* **2015**, *466*, 89–94, doi:10.1016/j.bbrc.2015.08.113.
